# Supplementary material for: A randomized trial of Adapted versus Standard versions the Transdiagnostic Intervention for Sleep and Circadian Dysfunction (TSC) implemented via facilitation and delivered by community mental health providers using train-the-trainer
Source: Res Sq. 2025 Jul 14:rs.3.rs-6414484. Preprint. [Version 1] doi: 10.21203/rs.3.rs-6414484/v1 (PMC12288530; doi:10.21203/rs.3.rs-6414484/v1)
Supplement: Supplement 1 [file NIHPPrs6414484v1-supplement-1.pdf]

Supplementary Files

This is a list of supplementary files associated with this preprint. Click to download.

- Gen2AdditionalFiles1to3March7editedtables.docx
- Gen2AdditionalFilesJune18FINAL.docx
- StaRIchecklistHarveyGen2.docx
